# Depressive Mood and Testosterone Related to Declarative Verbal Memory Decline in Middle-Aged Caregivers of Children with Eating Disorders

**DOI:** 10.3390/ijerph13030286

**Published:** 2016-03-04

**Authors:** Ángel Romero-Martínez, Nicolás Ruiz-Robledillo, Luis Moya-Albiol

**Affiliations:** Department of Psychobiology, University of Valencia, Avenida Blasco Ibañez, 2146010 Valencia, Spain; Nicolas.Ruiz@uv.es (N.R.-R.); Luis.Moya@uv.es (L.M.-A.)

**Keywords:** caregivers, declarative memory, depressive mood, eating disorders, testosterone

## Abstract

Caring for children diagnosed with a chronic psychological disorder such as an eating disorder (ED) can be used as a model of chronic stress. This kind of stress has been reported to have deleterious effects on caregivers’ cognition, particularly in verbal declarative memory of women caregivers. Moreover, high depressive mood and variations in testosterone (T) levels moderate this cognitive decline. The purpose of this study was to characterize whether caregivers of individuals with EDs (*n* = 27) show declarative memory impairments compared to non-caregivers caregivers (*n* = 27), using for this purpose a standardized memory test (Rey’s Auditory Verbal Learning Test). Its purpose was also to examine the role of depressive mood and T in memory decline. Results showed that ED caregivers presented high depressive mood, which was associated to worse verbal memory performance, especially in the case of women. In addition, all caregivers showed high T levels. Nonetheless, only in the case of women caregivers did T show a curvilinear relationship with verbal memory performance, meaning that the increases of T were associated to the improvement in verbal memory performance, but only up to a certain point, as after such point T continued to increase and memory performance decreased. Thus, chronic stress due to caregiving was associated to disturbances in mood and T levels, which in turn was associated to verbal memory decline. These findings should be taken into account in the implementation of intervention programs for helping ED caregivers cope with caregiving situations and to prevent the risk of a pronounced verbal memory decline.

## 1. Introduction

A chronic stress model is used to research caregivers of children and/or siblings diagnosed with a long-term psychological disorder. Chronic stress has been associated to impairments in verbal declarative memory of caregivers [1,2,3,4,5,6,7], which may be linked to functional outcomes and may have an impact on the ability to provide adequate care [8]. Several variables such as depressive mood, caregivers’ gender, and sex hormones have proved to mediate this cognitive decline [5,7,9,10,11]. The depression rate is higher in caregivers than in non-caregivers [1,2,6,7,12], being especially high in women, compared to men [13]. Depressive mood has been associated to caregivers’ verbal declarative memory decline [1,2,6,7], due to the variation in testosterone (T) levels [5,10,14]. 

The gradual and progressive decrease of serum T levels that occurs with aging in both men and women has been associated to verbal declarative memory decline [15,16]. This could be due to the high number of androgen receptors in the hippocampus, which is closely involved in memory processes [17]. In fact, it has been demonstrated that T maintains neurogenesis levels and supports synaptic density in the hippocampus, protecting the adult brain from degenerative diseases and memory decline [18]. T decrease due to age may accelerate the cognitive decline in healthy elderly men and post-menopausal women [15,19]. Moreover, aromatization of T to estradiol by aromatase, which modifies the effects of T on cognition, particularly in memory by reducing T levels, should also be considered [20]. Previous research indicates there is not a clear established association between T, gender and memory. However, higher endogenous serum T levels have been linked with better verbal memory performance in healthy elderly men and women [14,15,16,21]. Contrary to that, other studies show that T was negatively associated to verbal fluency, semantic memory, and episodic memory in women from 35 to 90 [21]. As a result, no relationship between T levels and declarative memory in middle-aged and elderly men and women has been reported [14,22,23]. Nonetheless, only one previous study analyzed the relationship between T and verbal declarative memory in a sample of women caregivers of children with autism spectrum disorders (ASD). This study revealed that T showed a curvilinear relationship with verbal memory performance, meaning that the increases of T were associated with the improvement in verbal memory performance, but only up to a certain point, as after such point T continued increasing and memory performance decreased [5].

To address these lacunae in literature, the main purpose of the current study was to evaluate the effect of chronic stress on verbal memory in middle-aged caregivers of children with eating disorders (ED), when compared with non-caregivers. It has been hypothesized that women had better declarative memory than men, although these differences are attenuated by age [24]. Moreover, caregiving is more harmful for women than for men, due to the fact that they present a higher sensitivity to develop psychiatric disorders in a caregiving situation [11]. Thus, we expected caregivers, particularly women, would show worse verbal memory performance than non-caregivers. As depressive mood has a mediating role in the relationship between caregiving and verbal declarative memory decline, especially in women [1,6,7,8,9,10,11,12,13,14,15,16,17,18,19,20,25,26,27], we expected to find that high depressive mood was associated to worse verbal memory performance, particularly in women. Finally, considering that the stressed middle-aged population and caregivers presented a higher T level average and a specific response to an acute laboratory stressor than non-caregivers [10,27], and the fact that T reduces the risk of cognitive decline in middle-aged men and women [15,16,17,19], we hypothesized that ED caregivers would present higher T levels at specific times in response to laboratory tasks than non-caregivers, and T would be positively related to declarative memory performance. 

## 2. Method

### 2.1. Participants

The final sample was comprised of 54 participants, 27 caregivers [fathers (*n* = 10) and mothers (*n* = 17)] of children with EDs, and 27 non-caregivers [fathers (*n* = 10) and mothers (*n* = 17)] who cared for normally developed children but not for any other person in the last two years. T data were incomplete for two parents of offspring with EDs and one non-caregiver, as the collected volume of saliva was insufficient to complete laboratory tests. Parents of children with EDs were recruited from a hospital day care center specialized in EDs (Valencia, Spain). Parents participated voluntarily in the study and signed a written informed consent in accordance with ethical principles regarding human research (Declaration of Helsinki) and approved by the University of Valencia Ethics Committee (code: H1360051962905). The age of the offspring with EDs ranged from 12–32 years (21.88 ± 5.48), with a sex ratio of 1 man to 10 women, according to the expected gender distribution in these disorders [28]. Children were clinically diagnosed with anorexia and/or bulimia by clinical staff, following the DSM-IV-R criteria. Inclusion criteria for participating in the study were as follows: being a first-degree relative of an individual with a clinically diagnosed ED, living in the same home as this individual, and being the main caregiver for at least the last two years before the study. An adequate control group was selected. It was comprised of non-adolescent fathers and mothers of healthy children without any chronic illness or dependence due to disability. Additionally, parents in the control group had not been caregivers for any relative with a chronic illness. For more details regarding caregivers and control group characteristics, see Table 1. 

### 2.2. Procedure

Participants were instructed to abstain from eating, drinking stimulants (such as tea, coffee, or alcohol), brushing their teeth, or smoking during the two-hour period before arriving at the laboratory. The experimental procedure was performed between 4:00 and 7:00 pm, in order to minimize hormonal variations attributable to the circadian rhythm. Each session lasted approximately two and a half hours. After participants arrived, anthropometric data (age, weight, height) were collected, and compliance with the instructions was checked. An initial preverbal memory test saliva sample was collected to measure T levels (baseline). Participants were then conducted to another noise insulated room with a constant temperature 22 ± 1 °C. After a habituation period, participants remained silent for three minutes and subsequent saliva samples were collected (to assess the preparation period for T levels). All participants spent the same time completing a verbal memory test. Immediately after completing the tasks, another saliva sample was collected (post-verbal memory test for T levels), while participants completed questionnaires for the evaluation of post-task anxiety and mood. Afterwards, participants returned to the first room where additional saliva samples were collected 20 and 30 min after the cognitive tasks were completed in order to assess T after 20 and 30 min, respectively. These times were carefully selected according to previous scientific literature on analyzing T fluctuations [5,27].

Finally, in a third session, participants completed the GHQ severe depression subscale. Moreover, in the case of caregivers, researchers conducted an interview regarding the characteristics of the care recipient (diagnosis, gender, age, autism quotient, global activity, and dependence percentage) and the status of the caregiver (years from the definitive diagnosis, time spent caregiving per week, burden, level of worry regarding the future and the disease).

### 2.3. Verbal Memory 

To measure declarative memory, the Spanish version of the Rey Auditory Verbal Learning Test (RAVLT) was used [29]. The RAVLT consisted of 15 nouns read aloud by the examiner (with a 1-s interval between each word) for five consecutive trials (Trials 1–5), each Trial was followed by a free-recall test. Patients were told that they would hear a list of 15 words and they should listen carefully because they would be asked to repeat as many as possible. The word presentation order remained fixed across the Trials. Instructions were repeated before each Trial to minimize forgetting. After a 20-min delay period, each subject was again required to recall the words (Trial 6). Other psychometric tests were carried out during the 20-min delay period. The previously described indices were computed [29,30,31], and are as follows: (1) correct responses given in each trial; (2) immediate recall score (the sum of all correct responses given in the five consecutive Trials); (3) verbal learning (difference between the number of words correctly recalled after the fifth and the first reading); (4) verbal forgetfulness (difference between the number of words correctly recalled after the fifth reading and at the delayed recall test, Trial 6). In addition, a percentage of forgetfulness (100x verbal forgetfulness/Trial 5) was also computed. 

### 2.4. Depressive Mood 

Depressive mood was assessed with a subscale “severe depression” of the shorter 28-item version of the General Health Questionnaire (GHQ-28) designed by Goldberg and Hillier [32]. Items were ranked on a 4-point Likert scale, from 0 (better than usual) to 3 (worse than usual). Cronbach’s alpha was higher than 0.92.

### 2.5. Hormonal Determination

Saliva was directly collected from the mouth and transferred to a glass tube to determine the levels of T. In fact, participants we asked to spit directly into the glass tube. Samples were frozen at −20 °C until analyzed by enzimoimmunoassay to determine the levels of T. Salivary T was quantified using the saliva T ELISA kit (Diagnostics Biochem Canada Inc, Dorchester, ON, Canada). Assay sensitivity was of 1 pg/mL and it was expressed in pmol/L. Intra and inter-assay variation coefficients of 3.98% and 7.98% were found, respectively. 

### 2.6. Data Analysis

After confirming the normality of the data using the Kolmogorov-Smirnov test, t-tests for each gender were performed with between-subject factors “group” for anthropometric data (age and body mass index or BMI). Chi-square statistics were calculated for the analysis of the frequencies of the demographic variables.

Considering T responses, the effectiveness of the memory test in the total sample was confirmed by the general linear model, repeated measures ANOVA, with “moment” (at five levels: for baseline, preparation, +0, +20 and +30) as a within-participant factor. To examine the group effects, a mixed-model ANOVA was conducted with “time” as the within-subject and “group” as the between-subject factors.

The magnitude of the T response was estimated by the area under the curve (AUC) with regard to the increase (AUC_i_), calculated using formulae derived from the trapezoidal rule, as previously described [33]. These formulae are simple additions of triangle and rectangle areas. AUCi was calculated with reference to the baseline measurement and it ignored the distance from zero for all measurements, and emphasized changes over time. Regarding hormonal parameters, AUC_i_ emphasized changes over time and was more related to system sensitivity [33].

For verbal memory performance, in order to explore “group” effects, a mixed-model ANOVA was performed with “moment” as the within-subject factor and ”group” as the between-subject factor. 

Greenhouse-Geisser corrections for degrees of freedom were applied when appropriate. For significant results, partial eta-squared was reported as a measure for effect size (η_p_^2^). Spearman or Pearson correlations were calculated to assess relationships between variables when appropriate for each group (caregivers and non-caregivers) and gender. Data analyses were carried out using SPSS 22.0 software (SPSS^®^ Statistics Inc. IBM Corp, Armonk, NY, USA). Statistical significance was defined as *p* values ≤ 0.05, while it was considered to be a significant tendency with *p* values from >0.05 to *p* ≤ 0.07. Average values are reported in tables as mean ± SEM.

## 3. Results

### 3.1. Participant Characteristics 

Caregivers did not differ from non-caregivers in age, BMI, menstrual cycle phases or socio-demographic variables. Moreover, no differences were found in baseline T levels. Nevertheless, women ED caregivers presented higher scores in the severe depression subscale of the GHQ than women non-caregivers (*t* (22.78) = 2.88, *p* = 0.008, *d* = 1.21) (see Table 1). 

### 3.2. T Response to the RAVLT

**Figure 1:**
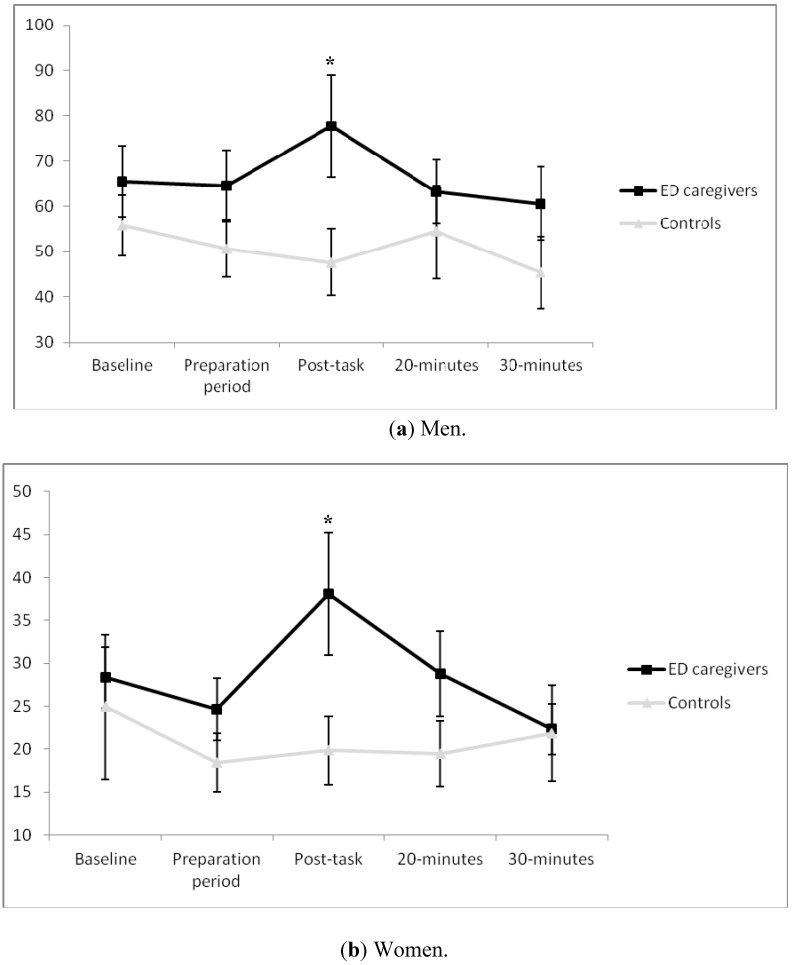
Testosterone levels (pmol/L) in ED caregivers and control groups for men (**a**) and women (**b**) * *p* < 0.05.

With regard to fathers of children with EDs, a significant “time × group” effect was found (ε = 0.66, *F*[2.67, 47.98] = 2.96, *p* = 0.047, η_p_^2^ = 0.14), with such fathers presenting higher T levels in post task than in the control groups (*p* < 0.05) (Figure 1a). Intra-group comparisons revealed a significant effect for “time” in men ED caregivers (*F*[1, 9] = 2.96, *p* = 0.033, η_p_^2^ = 0.25), with a significant increase from preparation to post task. Afterwards, a drop was observed from this point to 20 min post-task. Regarding the magnitude of response, there was a significant effect for “group” in AUC_i_ (t_18_ = 2.19, *p* = 0.042, *d* = 1.03). In fact, whereas T levels increased in women ED caregivers, they fell in the control groups (153.27 ± 458.58 and –338.67 ± 541.09, respectively).

Similarly, a significant effect for “time × group” interaction was found in the case of women (ε = 0.47, *F*[1.87, 57.95] = 3.27 , *p* = 0.048, η_p_^2^ = 0.10), showing higher post-task T levels in caregivers than in non-caregivers (Figure 1b). When analyzing the effect of the menstrual cycle, a significant effect was found for “time x phase of the menstrual cycle” interaction (ε = 0.47, *F*[1.87, 57.95] = 5.34, *p* = 0.03, n_p_^2^ = 0.13), although *post hoc* analysis did not reveal differences between menstrual cycle phases. In addition, the “time” factor was only significant in women ED caregivers (*F*[1, 16] = 3.15, *p* = 0.020, η_p_^2^ = 0.16). Specifically, T increased significantly from baseline to the preparation period. Afterwards, a significant decrease was observed from 20–30 min post task. Concerning the magnitude of response, a significant effect was found for “phase of menstrual cycle” (*F*[2, 44] = 3.50, *p* = 0.04, η_p_^2^ = 0.15), with a smaller T response in women with amenorrhea than in those in the follicular phase (*p* < 0.05 for all). Nevertheless, no significant differences were found between mothers of children with ED and control groups (104.56 ± 1507.25 and −344.82 ± 2148.31, respectively).

### 3.3. Verbal Memory (RAVLT)

**Figure 2:**
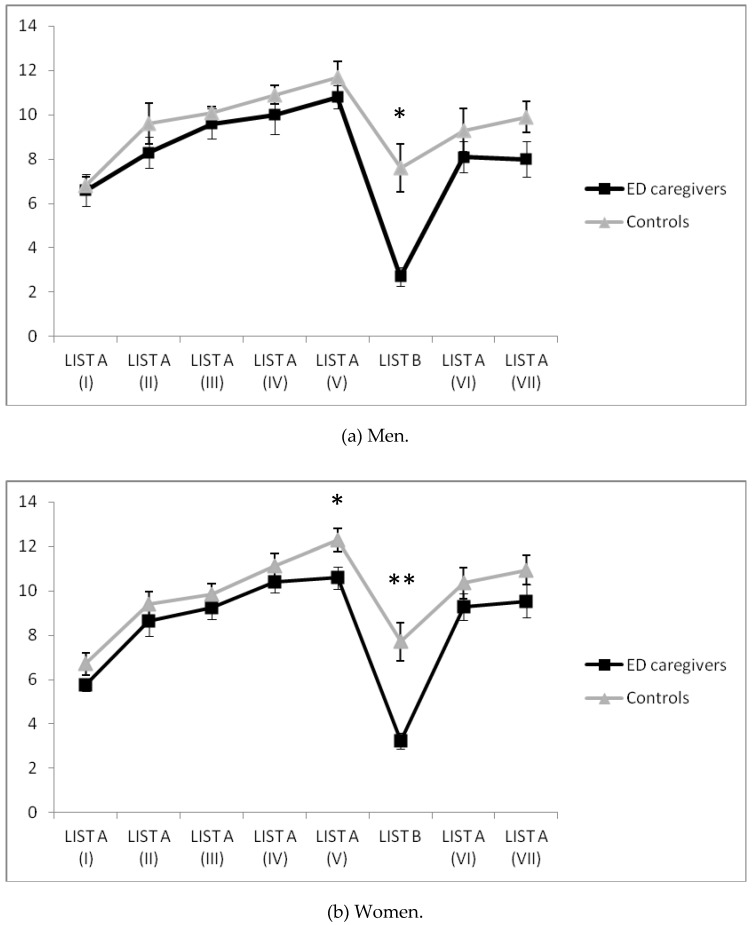
Verbal memory performance in ED caregivers and control groups for women (**a**) and men (**b**) * *p* < 0.05; ** *p* < 0.01.

Regarding men, a significant interaction “moment × group” effect was found in the RAVLT verbal memory learning test [*F*(3.68, 66.26) = 3.60, *p* = 0.012, η_p_^2^ = 0.17], with caregivers remembering fewer words on List B than non-caregivers (Figure 2a). 

With respect to women, “moment × group” interaction presented a significant effect in the RAVLT verbal memory learning test [*F*(4.68, 145.07) = 3.00, *p* = 0.015, η_p_^2^ = 0.09], with caregivers remembering fewer words on Lists A (V) and B than non-caregivers (Figure 2b). 

Finally, no significant differences between groups in recognition, immediate recall, verbal forgetfulness, percentage of verbal forgetfulness, verbal learning and learning curve were found. 

Calculation of the *post hoc* power calculation revealed an acceptable statistical power, which ranged from 60.9% to 83%, taking into account the probability of Type I Error (α), the endpoint means and the number of subjects. 

### 3.4. Relationships between Salivary T (baseline and AUCi), GHQ Severe Depression and Verbal Memory 

No significant relationships between variables were found in male participants. In women ED caregivers, high baseline T levels were related to a high number of remembered words on List A (I) and low scores in GHQ for severe depression (*r* = 0.766, *p* < 0.01, *r* = −0.495, *p* < 0.05, respectively). Moreover, high AUC_i_ T was associated to a low number of remembered words on List A (VI) and high scores in GHQ for severe depression, (*r*= −0.615, *p* < 0.01, *r* = 0.491, *p* < 0.05, respectively). Finally, GHQ for severe depression was associated to a low number of remembered words on List A (I) and high verbal forgetfulness (*r* = −0.722, *p* < 0.01, *r* = 0.727, *p* < 0.01, respectively). 

When analyzing women non-caregivers, a negative relationship between GHQ for severe depression and the number of remembered words on List A was found (V) (*r* = −0.514, *p* < 0.05).

The calculation of the statistical power of correlation models revealed a good statistical power, which ranged from 0.96 to 0.98, taking into account the probability of Type I Error (α), the sample size and the correlation coefficient observed. However, the relationships between T and depressive symptoms with a correlation coefficient inferior to 0.51 presented low statistical power (<0.80).

## 4. Discussion

ED caregivers remembered fewer words on List A (V)—especially women—and List B or interference (both) than non-caregivers. Women caregivers presented a higher depressive mood than women non-caregivers. Additionally, both women and men caregivers presented higher T levels and magnitude of response than non-caregivers. Particularly in women, depressive mood was associated to impaired verbal memory performance. Finally, only in women caregivers were high baseline T levels related to better verbal memory performance, but higher T responses to tasks were associated to worse verbal memory performance.

Most studies demonstrate that women caregivers are at a greater risk of verbal memory decline [1,2,4,5,6,7]. Our results confirm this hypothesis as women caregivers remembered fewer words on List A (V). They presented impaired performance over short delays (minutes), suggesting altered consolidation mechanisms. Nonetheless, these differences between groups disappear after long delays (25 min). Regarding the interference list, both men and women caregivers remembered fewer words than the control group participants. This could be explained by a high vulnerability to proactive interference, concretely a detrimental effect of prior learning when retaining new material. Thereby, these results can be explained by the difficulties in shifting attentional resources to the new verbal list [4]. Due to the similarity of the context, caregivers present an impaired skill to distinguish new from old material in comparison with the control groups. Our current results are in line with those of a previous study which analyzed the verbal declarative memory in women caregivers of children with ASD [5]. These caregivers presented higher sensitivity to proactive interference and verbal forgetfulness in comparison with non-caregivers. Nevertheless, it is interesting to note that in the current study caregivers presented a shorter history of caregiving (approximately 6 years) in comparison to caregivers in the previous study (approximately 10 years). Moreover, the care recipient was more independent and with a lower disability percentage in our study than in the previous one. However, caregivers in this study were slightly older (49 years) than those in the previous one (45 years) [5]. Thus, it seems that the deleterious effects of caregiving in declarative memory of caregivers are not affected by the disorder and the disability percentage of the care recipient. Accordingly, future studies should extend our results to a larger caregiver sample, with more caregiver types and more moderator variables in order to increase the external validity of the results. 

In line with previous research [1,2,7,12,13], these caregivers—especially women—reported a higher depressive mood than non-caregivers. Additionally, high depressive mood was associated to worse verbal memory performance in women caregivers. These results support the fact that depressive mood may affect memory decline in caregivers [7,9]. 

Previous studies show that chronic stress as is the case of caregivers present high T levels [5,10]. These high levels could be explained by an inverse relationship between T and cortisol (C) [34], due to the fact that caregivers tend to present a hypoactive hypothalamic-pituitary-adrenal axis response and low C levels [35]. In line with this, low basal T levels and high T increases were associated in our study to high depressive symptoms, which could be considered to be the opposite of the relation of C with depressive symptoms. This finding highlights the need to measure both hormones and analyze the relationship between them. Although the relationship between T and verbal memory is not clearly established [14,21,22,23], our results demonstrate that, particularly in women, T has a curvilinear relationship with verbal memory. In accordance with this, a previous study with women caregivers of children with ASD revealed the same relationship patterns between T and declarative memory [5]. These results reinforce the hypothesis that T keeps a curvilinear relationship with other cognitive abilities such as spatial cognition [36]. Due to the fact that T aromatization converts T to estradiol [20], future studies should consider T aromatization in order to analyze the curvilinear relationship between T and verbal memory in specific brain structures important for memory processes. Moreover, the role of the menstrual cycle should also be considered in larger samples in order to control its potential effects in T level fluctuations. 

The cross-sectional character of the study and the small sample size entail methodological limitations; hence, individual differences may mask other effects evaluated at one unique moment in life. Another limitation is the nonexperimental design; therefore conclusions cannot be made regarding causality. However, it is extremely difficult to conduct longitudinal studies regarding the caregiver’s personal situation. Moreover, it is important to note the possible existence of a neuropsychological endophenotype in eating disorders as revealed by a previous review study. This study describes patients with an eating disorder and their relatives as a population with a high cognitive rigidity and alterations in decision-making [37]. In line with this, it is quite possible that similarly, parents present particular memory or other neuropsychological features related to diathesis, rather than this being a result of chronic stress. However, future studies should consider this field of research in order to establish whether those group differences could be explained by chronic stress or by a cognitive endophenotype.

## 5. Conclusions 

Our results highlight that middle-aged caregivers of children with ED present a declarative memory decline, associated to depressive mood and T levels, particularly in women caregivers. This may mean that chronic stress alters mood and hormonal levels, which in turn increase the likelihood of accelerating normative declarative memory decline. These findings should be taken into account to understand the impact of chronic stress in impairments to acquire, integrate and remember information about concepts, objects and events, which may interfere with the normal functioning of caregivers and their ability to solve daily functioning problems, such as remembering medical and therapeutic instructions to provide optimal caregiving. This deleterious effect reinforces the importance for proper support and services to help caregivers cope with and reduce stress. Moreover, it is essential to develop neuropsychological intervention programs to target cognitive domains related to verbal processes.

## Figures and Tables

**Table 1:** Mean ± SD of age, BMI and demographic variables for women caregivers and non-caregivers.

Demographic Variables		ED Caregivers		Non-Caregivers	
		Men (*n* = 10)	Women (*n* = 17)	Men (*n* = 10)	Women (*n* = 17)
Age		47.90 ± 7.39	49.29 ± 4.41	42.70 ± 3.68	47.00 ± 2.57
BMI		29.56 ± 2.25	23.64 ± 4.03	28.97 ± 2.26	25.13 ± 4.46
Phases of menstrual	Luteal (1–14)	−	18%	−	35%
	Follicular (15-menstrual period)	−	41%	−	41%
cycle	Amenorrhea (>6 months)	−	41%	−	24%
Educational level	<12 years	30%	41%	30%	29%
	College degrees	70%	59%	70%	71%
Marital status	Married/Cohabiting	70%	82%	100%	94%
	Divorced/Widow/Single	30%	18%	0%	6%
Employment status	Employed	80%	88%	80%	88%
	Unemployed	10%	0%	10%	0%
	Retired/other	10%	12%	10%	12%
Number of children		2.54 ± 2.15	2.17 ± 1.82	1.77 ± 0.72	2.18 ± 0.85
GHQ severe depression		2.10 ± 4.60	3.41 ± 3.04	0.60 ± 1.26	1.06 ± 1.43
Caring years from definitiveDiagnosis		6.38 ± 3.55	5.83 ± 4.14		
Global activity		61.50 ± 15.06	60.79 ± 10.68		
Independence (Barthel index)		100 ± 0.00	99.06 ± 5.91		
Disability percentage		3.30 ± 10.44	0.00 ± 0.00		
